# Occurrence of *Pseudomonas syringae* pvs. actinidiae, actinidifoliorum and Other *P. syringae* Strains on Kiwifruit in Northern Spain

**DOI:** 10.3390/life14020208

**Published:** 2024-01-31

**Authors:** Ana J. González, David Díaz, Marta Ciordia, Elena Landeras

**Affiliations:** 1 Servicio Regional de Investigación y Desarrollo Agroalimentario (SERIDA), Ctra. AS-267, PK 19, 33300 Villaviciosa, Spain; dcolinas@serida.org (D.D.); mciordia@serida.org (M.C.); 2 Laboratorio de Sanidad Vegetal del Principado de Asturias, C/Lucas Rodríguez Pire, 4-Bajo, 33011 Oviedo, Spain; elena.landerasrodriguez@asturias.org

**Keywords:** bacterial canker, *Actinidia deliciosa*, phytopathogenic bacteria, Multilocus Sequence Analysis (MLSA), phylogenetic tree

## Abstract

*Pseudomonas syringae* pv. actinidiae (Psa), the agent causing bacterial canker of kiwifruit, has been present in the Principality of Asturias (PA), Northern Spain, since 2013, although with restricted distribution. In this study, 53 strains collected in kiwifruit orchards in PA during the period 2014–2020 were characterized by a polyphasic approach including biochemical and phylogenetic analysis. Thirty-three strains, previously identified by PCR as Psa, have been found to be a homogeneous group in phylogenetic analysis, which seems to indicate that there have been few introductions of the pathogen into the region. Two strains were confirmed as *P. syringae* pv. actinidifoliorum (Pfm), so this is the first report of Pfm in the PA. The remaining 18 strains were found to be close to *P. avellanae* and *P. syringae* pv. antirrhini or to strains described as Pfm look-alikes. Pathogenicity tests carried out on peppers with a selection of strains have shown that both Psa and Pfm caused clear damage, while the 18 atypical strains caused variable lesions. It would be necessary to carry out pathogenicity testing of atypical strains on kiwifruit plants to study the role of these strains in the kiwifruit pathosystem to evaluate their pathogenic potential in this crop.

## 1. Introduction

Kiwifruit (*Actinidia deliciosa* (Chev.) Liang and Ferguson), although originally from China, is now cultivated in many different zones of the world, including in New Zealand, Chile, the USA, Korea, Japan and several European countries (Italy, Greece, France, Spain and Portugal). In Spain, kiwifruit cultivation was first introduced in the northwest region at the end of the 1960s. In the Principality of Asturias (PA), which is situated in the center of Spain’s northern coast, cultivation of the fruit was not established until 1972–1973, and it was not until the 1980s that kiwifruit orchards became relatively widespread. The latest data highlight the PA as the second largest kiwifruit producer in Spain, with a total of 280 ha producing up to 3823 t. These data make kiwifruit the second most important commercial fruit crop in this region, preceded only by apple (cider and dessert apple) with a total production of 13,086 t, despite variations due to the pronounced biannual alternation of this species [1].

Diseases have been widely observed in this crop. Due to the recent domestication of kiwifruit, in clear contrast with other, more established crops (e.g., vineyards or apples), the emergence of fungal and bacterial agents affecting kiwifruit plants was documented once commercial kiwifruit orchards became significant [2].

Among its bacterial pathogens, *Pseudomonas viridiflava* and *P. syringae* pv. syringae cause leaf spots and bud and flower rot that decrease production [3,4,5,6]. *P. syringae* includes more than 60 well-characterized pathovars including two that have been described as pathogenic agents specific to this host: *P. syringae* pv. actinidiae (Psa) and *P. syringae* pv. actinidifoliorum (Pfm) cause bacterial canker and bacterial spots, respectively [7,8,9]. Bacterial canker is the most damaging disease at present; it is considered the greatest risk to kiwifruit culture and is responsible for both production and plant losses.

Psa was first described in Japan in 1984 as the causal agent of the bacterial canker of kiwifruit affecting *Actinidia* spp. [7]. Afterwards, it was detected in China in 1989 [10] and Korea in 1992 [11]. In Europe, the first report was in Italy in 1992 [12], with a subsequent and more important epidemic outbreak in 2008 [13]. Since then, the disease has spread quickly to other countries: Turkey in 2009 [14], Portugal and Chile in 2010 [15,16], France, Spain, Switzerland and New Zealand in 2011 [17,18,19,20,21], Georgia and Slovenia in 2013 [22,23], Greece in 2014 [24] and Argentina in 2015 [25].

Different biovars of Psa are dispersed around the world and were initially classified on the basis of the multilocus sequence analysis (MLSA) method, the detection of type III secretion system effector genes and the presence of phaseolotoxin and/or coronatine phytotoxins [26,27,28,29,30]. Biovar 1 (Psa 1) includes strains detected in Japan that produce phaseolotoxin; biovar 2 (Psa 2) includes Korean strains that produce coronatine; biovar 3 (Psa 3) includes the Italian aggressive strains that do not produce phytotoxins and were responsible for the 2008 epidemic outbreak; biovar 4 (Psa 4), currently considered by Cunty et al. [9] to be a new pathovar, *P. syringae* pv. actinidifoliorum (Pfm) which includes low virulence strains; biovar 5 (Psa 5) includes Japanese endemic strains isolated in 2012 without producing phytotoxins; and biovar 6 (Psa 6), found in Japan in 2015, could also be an endemic lineage similar to biovar 5 but differs in that it produces phytotoxins, phaseolotoxin and coronatine [29,31].

Among the different Psa biovars, the most virulent is biovar 3, also named Psa-V, causing severe damage in kiwifruit orchards worldwide [9,10,27,32]. In 2013, McCann et al. [2] sequenced the genome of more than 30 Psa strains, concluding that Psa-V isolates from different sources are very similar in phylogenetic terms, with the exception of a Chinese isolate which is also virulent. Currently, three different clades are considered in Psa with inter- and intra-clade pathogenic variability [33].

This group of virulent bacteria is of worldwide interest as a model of study, as it is a disease that has been described recently, which allows its diversification and evolution patterns to be studied [2]. Furthermore, its great ease of dissemination, the difficulty of controlling it and the considerable crop damage it causes are other reasons for subjecting it to detailed investigation.

In Europe, Psa has been included in the EPPO (European Plant Protection Organization)’s A2 list of pests recommended for regulation as quarantine pests since 2012 [34]. In Spain, bacterial canker affecting *Actinidia* spp. was reported for the first time in Galicia [18,19]. Official information shows that Psa was detected in PA in 2013; later, an outbreak detected in the Basque Country was eradicated in 2015. Psa was also detected in Navarra in 2018, and in 2019, it was again detected in the Basque Country and Asturias, with the infected plants being destroyed in the Basque Country. In 2020, two outbreaks were detected, one in Asturias and the other in Navarra [35,36,37,38,39]. In addition, Pfm was identified in Galicia [40], and a recent study [41] cites the presence of a bacteria actinidifoliorum look-alike, pending confirmation, in Spain, although without specifying the region.

In this study, we present the characterization and identification by means of biochemical and genetic approaches of Psa and other related *P. syringae* strains isolated from kiwifruit orchards in PA over the years 2014–2020. The results obtained provide information about the taxonomy and phenotypic variation of the strains involved in kiwifruit canker, which will be useful for further epidemiological studies and for strengthening biosecurity strategies in the management of bacterial pathogens in kiwifruit orchards.

## 2. Materials and Methods

### 2.1. Bacterial Isolates and Phenotypic Characterization

#### 2.1.1. Bacterial Isolates

A total of 53 bacterial isolates obtained from samples of kiwifruit analyzed between 2014 and 2020 in the PA, specifically on *A. deliciosa* cv. ‘Hayward’ from commercial kiwifruit orchards and one domestic orchard, as well as one on cv. ‘Tomuri’ male vines, were investigated in the present study. Of these, thirty-three had previously been identified as Psa according to EPPO Diagnostic Protocols PM7/120(1) [42], eighteen of which were isolated in 2014, six in 2015, three in 2018 and the remaining six in 2020. In addition, 20 strains isolated in 2014 were included in this study, since they amplified one of the two bands expected in the PCR test developed by Gallelli et al. [43] and the single band expected in the PCR developed by Rees-George et al. [44].

Bacterial isolates were coded and preserved by two different methods, at −80 °C with 50% DMSO (Dimethyl Sulfoxide), and freeze-dried, in the collection of the Phytopathology Laboratory of SERIDA (LPPA).

#### 2.1.2. Phenotypic Characterization

A total of 25 tests were performed with all the isolates: Gram; fluorescence on King B medium; glucose oxidation; growth at 36 °C; levan production; oxidase; arginine dihydrolase; hypersensitivity in tobacco leaves; esculin, gelatin, casein and tween 80 hydrolysis; use of sucrose, adonitol, d-tartrate, l-lactate, trigonelline, betaine, homoserine, quinate and xylose in Ayer’s medium and mannitol, erythritol, sorbitol and inositol in Hellmers’s broth as sole carbon sources [45,46,47,48].

### 2.2. Detection of Genes Involved in Toxins and Levan Production

#### 2.2.1. Detection of Toxin Genes

Fragments of the *tox-argK* gene cluster involved in the biosynthesis of the phaseolotoxin and the *cfl* gene encoding coronatine were amplified [49,50,51]. As controls, *P. syringae* pv. tomato CECT (Spanish Type Culture Collection) 126, LPPA 696, *P. syringae* pv. phaseolicola LPPA 597 and LPPA 1407 were used.

To complete the study, the detection of *syrB/D* and *sypA* genes involved in syringomicin and syringopeptin synthesis, respectively, were included [52,53,54]. As positive controls, strains CECT 4429 and LPPA 56 were used.

#### 2.2.2. Detection of Levansucrase Genes

The analysis of the distribution of *lsc* genes in the isolates under study was carried out by PCR amplification with specific primers. *lscA/B/C* genes were analyzed using LPPA 28 and LPPA 275 as controls, respectively [55].

### 2.3. Amplification of Housekeeping Genes

Three genes were amplified: the *gltA* gene, also known as *cts* encoding citrate synthase, *rpoD*, encoding RNA polymerase sigma factor and *gyrB*, encoding the B subunit of DNA gyrase [56,57]. The amplified fragments were sequenced by Secugen (Spain) or Eurofins (Germany). Sequences were submitted to GenBank and the accession numbers are shown in Supplementary Table S1. The sequences were compared with those deposited in databases through the BLAST algorithm [58].

### 2.4. Phylogenetic Analysis

Sequences of concatenated and individual amplified genes were aligned using Clustal W [59], and phylogenetic trees were constructed based on each individual locus and the three concatenated genes by the maximum likelihood method with the Tamura–Nei model. Their topological robustness was evaluated by bootstrap analysis based on 1000 replicates using MEGA 11 software [60]. *Pseudomonas asturiensis* LPPA 221^T^ was used as the outgroup. Sequences of Psa, Pfm and other pvs. of *P. syringae* obtained from the GenBank databases were included for comparison in the phylogenetic analysis (Supplementary Table S1).

The number of segregating sites (S) and the mean of the nucleotide diversity (π), defined as the average number of nucleotide differences by site between sequences of the whole population, were calculated both for the individual genes and the concatenated sequences, also using the Kimura two-parameters model in MEGA 11. Data on the evolutionary divergence of Psa–Pfm and strains of *P. syringae* are shown in Supplementary Tables S2 and S3 [60].

### 2.5. Pathogenicity Assays

Pathogenicity was tested by inoculation of the isolates on marketable fruits of *Capsicum annuum* cv. ‘California’ according to Abelleira et al. [40]. Twelve LPPA strains (2727, 2983, 3697, 3700, 2668, 2669, 2723, 2725, 2757, 2758, 2759, 2760) were selected for the assay. One pepper per strain, previously cleaned with ethanol, was inoculated by ten 2 mm punctures made in the epidermis and then filled with 15 µL of a bacterial suspension of 10^8^ cfu/mL. Strains NZ 10627 and CFBP 8039 as positive controls and 15 µL of Luria broth as negative control were used. Inoculated peppers were kept at 25 °C for 7 days. Koch’s postulates were fulfilled by identification of the re-isolated bacteria from the symptomatic lesions on inoculated peppers.

## 3. Results and Discussion

### 3.1. Bacterial Isolates and Phenotypic Characterization

#### 3.1.1. Bacterial Isolates

Bacterial strains recovered over 2014–2020 in several kiwifruit orchards in PA were first grouped into Psa (33 strains) and atypical strains (20 strains) according to the results of the two PCR tests performed. Atypical strains amplified the expected band (280 bp) in the simple PCR [44] but only the upper one (492 bp) in the duplex PCR test [43]. This result is consistent with that described for Pfm [9]; however, our study indicates that only two out of twenty strains can be assigned to this pathovar. Data for the total of 53 strains are shown in Table 1.

Strains LPPA 2983–2985 were isolated from *A. deliciosa* cv. Tomuri, while all others were isolated from *A. deliciosa* cv. Hayward.

The presence of Psa in Asturias was only notified by the Plant Health Service of the Government of PA in 2013 in the municipalities of Pravia and Langreo [35]. Some other locations have been added since then: the area of Salas in 2018 [37], the area of Carreño in 2019 [38] and Piloña and Villaviciosa in 2020 [39]. However, there is no indexed bibliography publishing these results except for the recent reference by Moran et al. [41]. These authors describe new genetic lineages of a *P. syringae* pv. actinidifoliorum look-alike isolated from the main kiwifruit-producing areas in the north and east of Spain, although without providing details of the locality of origin of the strains under study and without revealing the presence of either Psa or Pfm in the series they collected. Therefore, this is the first article in which the strains collected in the PA have been studied and in which isolates of both Psa and Pfm have been found.

#### 3.1.2. Phenotypic Characterization of the Isolates

All the strains analyzed were negative for Gram stain, oxidase and arginine dihydrolase and positive for hypersensitivity on tobacco leaves, oxidation of glucose and also the use of sucrose and xylose as sole carbon sources.

The use of sucrose as a source of carbon rules out that any of the atypical strains correspond to *P. viridiflava*, which, as was mentioned in the introduction, is one of the bacteria responsible for damage in kiwifruit. Typical *P. viridiflava* strains are levan negative, but strains of these bacteria isolated from kiwifruit and other crops have been described in Asturias as levan positive. However, these levan-positive strains remain negative when utilizing sucrose as the sole carbon source [6].

Concerning the Psa group, phenotypic characterization evidenced that the series under study shows differences with those described by other authors in several characteristics, such as the use of xylose, trigonelline, mannitol, sorbitol and inositol as the only source of carbon and casein, tween 80, gelatine and esculin hydrolysis [7,11,13]. However, Abelleira et al. [40] have already found a virulent strain of Psa that was esculin positive.

The LPPA 2768 and LPPA 2769 strains have been identified in the present study as Pfm on the basis of the biochemical and physiological tests, as both showed fluorescence and were esculin positive.

The Ps group showed greater heterogenicity. However, all of them were esculin and gelatin positive, and only one strain was not fluorescent. Biochemical features of the isolates under study are compiled in Table 2.

These results would emphasize the hypothesis that at least most of the atypical strains collected in 2014 would not correspond to Psa. Furthermore, fluorescence appeared to be a key factor for selecting Psa strains, since 100% of those identified as such were nonfluorescent compared to 5% of the atypical strains.

### 3.2. Phytotoxin and Levansucrase Gene Detection

#### 3.2.1. Phytotoxin Gene Detection

None of the 53 strains studied amplified the gene fragments corresponding to phaseolotoxin or coronatine toxins, whereas the reference strains *P. syringae* pvs. tomato and phaseolicola were found to be positive. These results are consistent with what is expected for virulent Psa strains corresponding to biovar 3.

Phytotoxins were considered the major virulence determinants in *P. syringae* [54] and frequently their production increases plant damage. Coronatine and phaseolotoxin generally induce chlorosis, whereas syringomicin and syringopeptin cause necrosis [61].

None of the 53 strains amplified *syrB*, *syrD* or *sypA*. On the basis of these results, we would rule out that any of the atypical strains are *P. syringae* pv. syringae, which is another of the bacteria described as pathogenic in kiwifruit [4,5].

#### 3.2.2. Levansucrase Gene Detection

Levan production is a significant taxonomic feature of the fluorescent *Pseudomonas* group to which *P. syringae* belongs and is considered a virulence factor in diseases caused by phytopathogenic bacteria [62]. Levan is a high-molecular-weight polysaccharide synthesized by several levansucrase isoenzymes (Lsc), so the presence of genes involved in the production of these enzymes was studied. The *lscB* gene is located in a plasmid and produces an extracellular enzyme, and the *lscC* gene is positioned on the chromosome and its product is a periplasmic levansucrase, while *lscA* is considered a cryptic gene with chromosomal location and is not related to levansucrase activities [55]. It has been suggested that *lscA* may be an ancestral levansucrase gene that lost the ability to express the enzyme in *P. syringae* [63].

All Psa strains amplified at least one of the *lsc* genes, specifically *lscC* (18 strains, 54%) or both *lscB* and *C* (15 strains, 45%), which is consistent with levan production. By contrast, none of the Psa strains amplified the *lscA* gene.

Neither of the two strains of Pfm amplified any of the *lsc* genes even though they produced levan, so the existence of additional levansucrase isoenzymes cannot be ruled out, as has already been mentioned in previous work with other *P. syringae* strains [64].

Moreover, in the atypical strain series, we have found all the possible combinations, which means bacteria with and without *lsc* genes and producing or not producing levan. Six out of eighteen atypical strains (33%) amplified *lscC*, but only one of them, LPPA 2759, is also levan positive, so it is possible that the gene was not functional in the remaining five. Two strains (11%) amplified both *lscB* and *C* genes and were levan positive. A total of 50% of the strains in this group did not amplify the three screened genes, but LPPA 2758 was positive for levan production. In the latter case, and as previously said for Pfm, it is possible that isoenzymes other than those tested are involved [65].

### 3.3. Multilocus Sequencing Analysis (MLSA)

The majority of the strains identified as Psa had at least 99% similarity to the deposited Psa sequences when compared to the sequences of each gene by BLAST.

This was true for the *gltA* gene, except for strain LPPA 2980, which had a similarity percentage of 98.77%. With reference to the *gyrB* gene, the similarity percentage was >99%, except in the case of LPPA 2717 and LPPA 2718, for which the values were 98.40% and 98.45%, respectively. In contrast, the *rpoD* region of all the strains identified as Psa shared more than 99% sequence similarity with the deposited Psa sequences.

Sequences of *gltA*, *gyrB* and *rpoD* from LPPA 2768 and LPPA 2769 have 100% similarity with *P. syringae* pv. actinidifoliorum strain ICMP 18803, corresponding to lineage 1 of this pathovar.

In the heterogeneous group of eighteen atypical strains, in the case of the *gltA* gene, for ten strains, the relatively closest matches were Psa and *P. avellanae*; in three strains, the closest matches were cf. Pfm and *P. avellanae*; in another three, it was cf. Pfm and in two others, it was *P. syringae* pv. tomato. With *gyrB*, eleven strains had *P. avellanae* as their closest relative, five had cf. Pfm and *P. avellanae* and two had *P. syringae* pv. tomato. Finally, in the case of *rpoD*, ten strains had *P. avellanae* as the closest match, two had cf. Pfm and *P. avellanae*, four had cf. Pfm and *P. syringae* pv. tomato, while two were closest to *P. syringae* pvs. antirrhini and tomato.

### 3.4. Phylogenetic Trees

**Figure 1:**
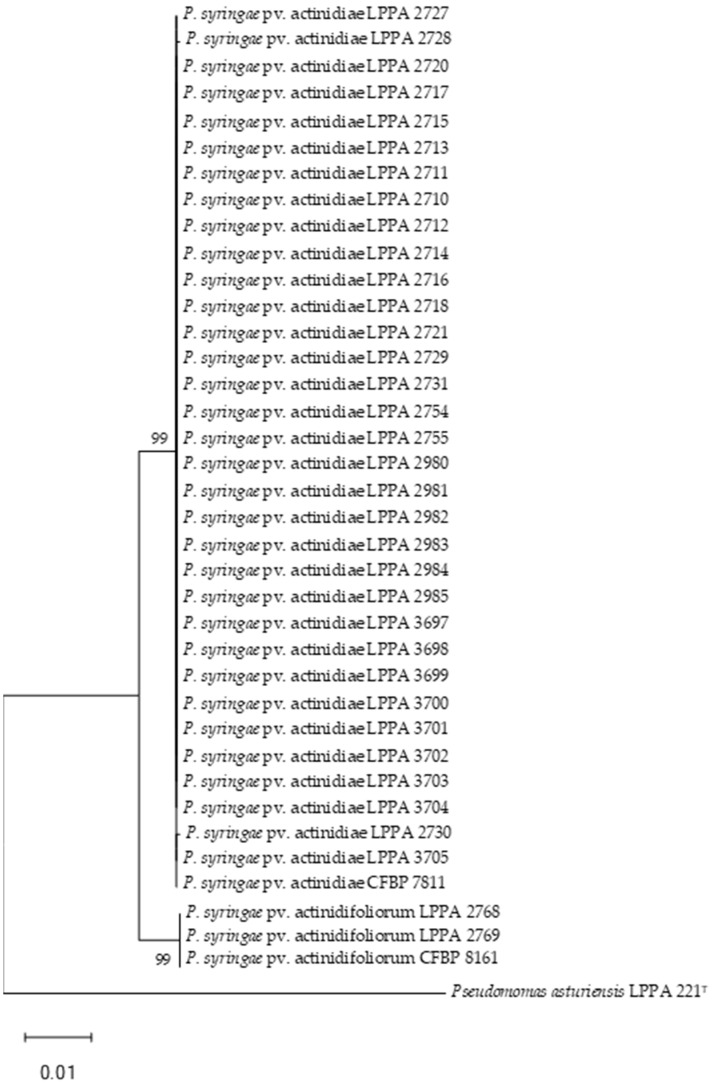
Phylogenetic tree of Psa and Pfm strains based on concatenated partial sequences of the *gltA*, *gyrB* and *rpoD* genes by using the maximum likelihood method and Tamura–Nei model. Bootstrap values ≥ 50% (1000 replicates) are indicated at branch points. Bar scale, substitutions per site. This analysis involved 38 nucleotide sequences, with a total of 2263 positions. Analyses were conducted in MEGA11 [60]. *P. asturiensis* LPPA 221^T^ was used as outgroup and *P. syringae* pv. actinidiae CFBP 7811 and *P. syringae* pv. actinidifoliorum CFBP 8161 as controls. Accession numbers of the sequences are shown in Supplementary Table S1.

Phylogenetic trees generated with the concatenated sequences of the *gyrB*, *gltA* and *rpoD* genes of the Psa and Pfm strains (Figure 1) involved 2263 bp (879 *gltA*, 603 *gyrB* and 781 *rpoD*). The analysis based on each of the genes separately (Supplementary Figure S1A–C) allows the differentiation of Psa and Pfm from the atypical strains. Estimates of the evolutionary divergence between the Psa and Pfm sequences are shown in Supplementary Table S2.

As shown in Figure 1, all isolates previously identified as Psa clustered with Psa strain CFBP 7181 with a 99% bootstrap. On the other hand, two strains of those considered atypical Psa, as they did not amplify the 230 bp band in the duplex PCR, clustered with CFBP 8161 corresponding to Pfm lineage 1. This pathovar was previously referred to as Psa biovar 4 and was subsequently considered a new pathovar [9]. The fact that this pathovar only amplified the 492 bp band of the two expected for Psa in duplex PCR had been previously described by Cunty et al. [9], but in our case, 18 more strains showed this result, although they were not clustered with Pfm. This implies that both the simple and duplex PCR allow the identification of Psa but not of Pfm.

The numbers of segregating sites (S) and the nucleotide diversity (π) of the concatenated sequence of the three housekeeping genes (*gltA*, *gyrB* and *rpoD*) with respect to the three individual genes are shown in Table 3. *GyrB* was the gene that contributed most to nucleotide diversity, a result that concurs with that already obtained by Yin et al. [65]. However, *gltA* was the gene that contributed the least to nucleotide diversity, both at the individual level and with the three concatenated genes, *rpoD* being the one that is closest to the value of π calculated for the three concatenated genes.

The Psa strains analyzed seem to be a homogeneous group, as we had previously assumed since the plants analyzed in 2014 had only two origins: Galicia, which is the first Spanish geographic area where the disease appeared [18], and Italy, which is where the outbreak produced by Psa biovar 3 was described [13].

**Figure 2:**
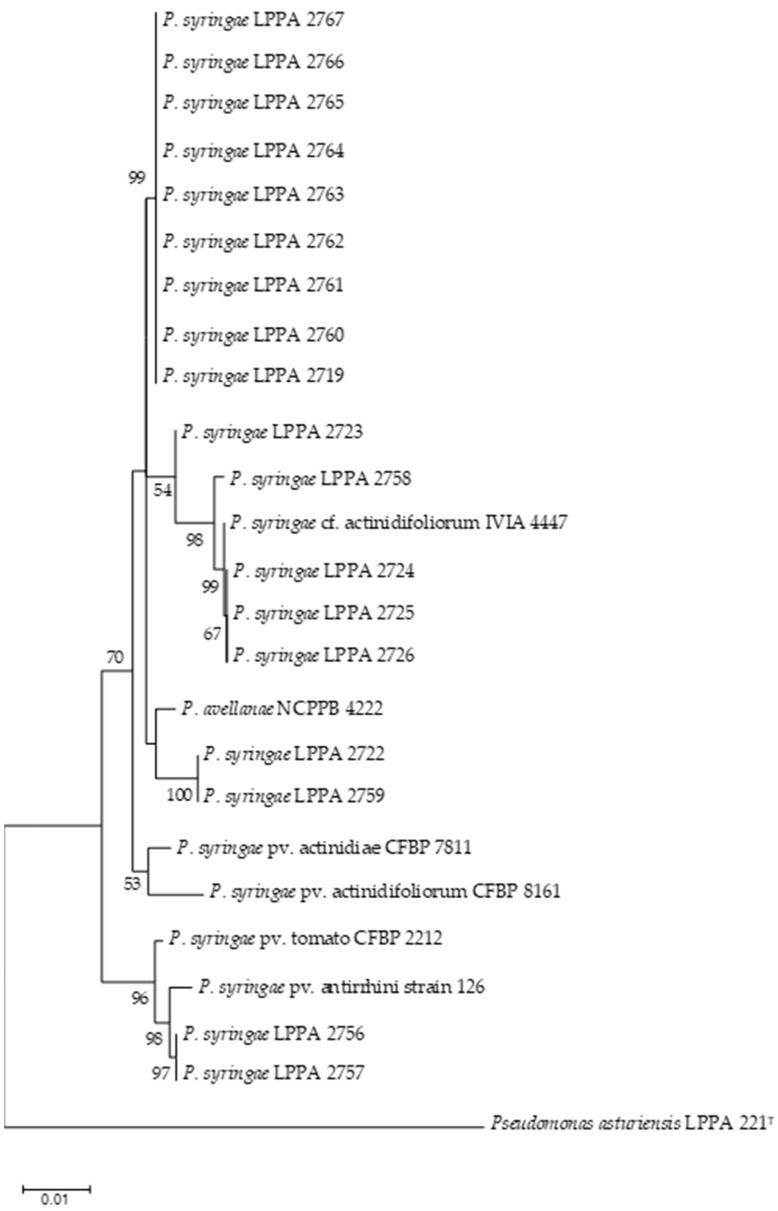
Phylogenetic tree of Ps strains based on concatenated partial sequences of the *gltA*, *gyrB* and *rpoD* genes by using the maximum likelihood method and Tamura–Nei model. Bootstrap values ≥ 50% (1000 replicates) are indicated at branch points. Bar scale, substitutions per site. This analysis involved 25 nucleotide sequences, with a total of 2092 positions. Analyses were conducted in MEGA11 [60]. *P. syringae* pv. actinidiae CFBP 7811, *P. syringae* pv. actinidifoliorum CFBP 8161, *P. syringae* pv. tomato CFBP 2212, *P. avellanae* NCPPB 4222 and *P. syringae* pv. antirrhini strain 126 were included as controls and *P. asturiensis* 221^T^ as outgroup. Accession numbers of the sequences are shown in Supplementary Table S1.

This result contrasts with the findings of other authors in other geographical areas, showing high genetic variability in Psa, whether related to the origin of the strains [66] or not [67]. In Europe, it seems that the clonal expansion of Psa was followed by a broad genomic diversification, as reported by Figueira et al. [68]. In Portugal, the presence of two genetically distinct subpopulations of Psa biovar 3 has been recently described [69,70]. In the phylogenetic trees made with the 18 atypical strains, sequences of the pathovars that were found to be most closely related by BLAST were included. Figure 2 shows the phylogenetic tree based on concatenated partial sequences of the *gltA*, *gyrB* and *rpoD* genes, and the trees with the individual genes are included in Supplementary Figure S2A–C. Estimates of evolutionary divergence between atypical strain sequences are shown in Supplementary Table S3.

As can be seen in Figure 2, nine strains were grouped into a cluster with a bootstrap of 99%. Three strains isolated from kiwifruit samples collected in the same orchard were grouped with the IVIA 4447 strain that had been deposited as unconfirmed (cf) Pfm. We cannot know if the geographical origin of these strains is the same since Morán et al. [41] did not specify this data in their article, in which they only refer to “isolates from Asturias and other areas of Spain”. In addition, we have not confirmed them as Pfm since they did not group with the Pfm control strain CFBP 8161. Two strains isolated from the same orchard and year, LPPA 2722 and LPPA 2759, were grouped with *P. avellanae* as the closest. LPPA 2756 and LPPA 2757, also from the same orchard and year, were grouped with *P. syringae* pv. antirrhini as the closest. None of these strains have fully matched any of the nearby pathovars.

Concerning nucleotide diversity (Table 4), it can be observed that the gene that contributed the least to nucleotide diversity was *gltA*, while that which contributed the most was *gyrB*. However, *rpoD* was the gene that showed a value of nucleotide diversity closest to that obtained with the set of the three concatenated genes. This result matches with that obtained for Psa in the studied series (Table 3) and confirms that *rpoD* is a good phylogenetic marker, as described by other authors [71].

### 3.5. Pathogenicity Assays

**Figure 3:**
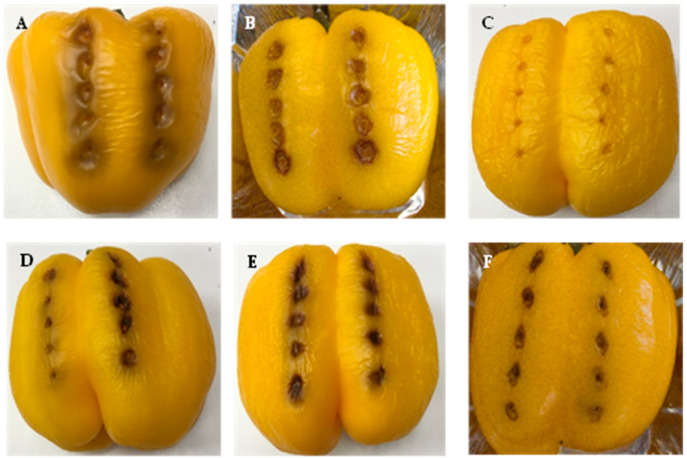
Inoculation of Psa and Pfm on *Capsicum annum* cv. California. (**A**) LPPA 2769. (**B**) Pfm CFBP 8039. (**C**) Control without inoculation. (**D**) LPPA 2727. (**E**) LPPA 3700. (**F**) Psa NZ 10627.

Necrotic areas were observed in the pathogenicity tests carried out on yellow peppers (*Capsicum annum* cv ‘California’) inoculated with representative strains of Psa (LPPA 2727, LPPA 3700, NZ 10627E) and Pfm (LPPA 2769, CFBP 8039), without finding major differences between the Asturian strains; the Psa control strain presented the least browning. Control fruits showed no lesions (Figure 3). The inoculated bacteria were recovered and reidentified from the lesions produced.

**Figure 4:**
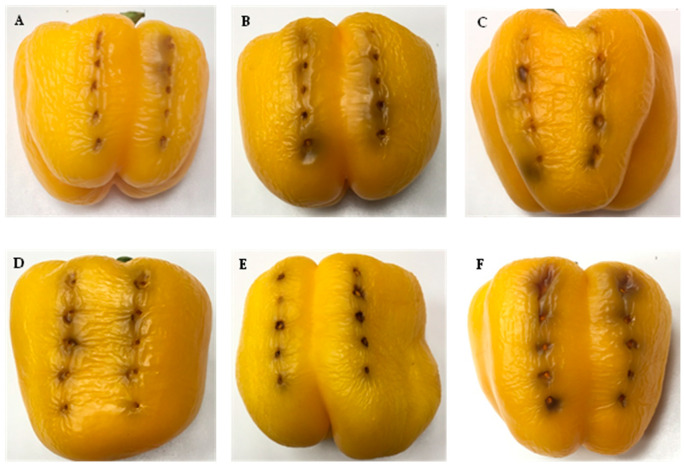
Inoculation of atypical strains in *Capsicum annum*. (**A**) LPPA 2723. (**B**) LPPA 2725. (**C**) LPPA 2757. (**D**) LPPA 2758. (**E**) LPPA 2759. (**F**) LPPA 2760. Control is shown in Figure 3C.

The results of the inoculations with the strains selected from the group of atypical strains (LPPA 2723, LPPA 2725, LPPA 2757, LPPA 2758, LPPA 2759, LPPA 2760) are shown in Figure 4. The damage caused by these bacteria is slightly less than that caused by Psa and Pfm.

When comparing lesions on peppers from Figure 3 and Figure 4, slight differences in the inoculation results are observed, with those in Figure 3 being more intense. This result suggests that it is possible that these atypical bacteria have low virulence and therefore may cause mild damage to kiwifruit, so the economic impact would be milder on the kiwifruit crop than that caused by Psa.

The important contribution that Abelleira et al. [40] made by testing different hosts in their inoculations has allowed us to work with pepper, which has several advantages: its lower acquisition and maintenance cost, the availability of the material to be inoculated at any time of the year and the greater speed in obtaining the results.

Further work is needed to identify atypical strains by analyzing the whole genome.

On the other hand, it is necessary to clarify the role played by these atypical bacteria in the symptoms observed in the orchards. *Pseudomonas syringae* is found in different environments; it has been isolated from cultivated and wild plants, as well as in weeds [72,73,74,75,76], but also in the environment, e.g., in snow, clouds, soil, etc. [77,78,79]. So, it is necessary to clarify the role that *P. syringae* strains isolated from kiwifruit may have in the kiwifruit pathosystem. From what has been indicated in this study, it seems that these strains could be of low virulence, at least in the cv. Hayward, but it would be advisable to carry out inoculations on kiwifruit plants in order to follow the development of the symptoms and determine if any of them may be of importance to kiwifruit culture and, therefore, require some type of treatment.

However, it should be noted that the treatment of bacteriosis occurring in agriculture is difficult. Nowadays, many studies are being carried out in order to find a treatment that can mitigate the damage caused by Psa, bearing in mind that the use of antibiotics is restricted for health reasons as part of the EU’s “One Health” strategy. Therefore, efforts are directed towards the development of biological control agents (BCAs). In this context, phages that infect the bacterium have been described to control this disease [80,81,82,83,84,85].

## 4. Conclusions

1. The presence in the Principality of Asturias of *P. syringae* pv. actinidiae has been confirmed, and *P. s*. pv. actinidifoliorum has been detected for the first time.
2. The Psa strains under study have been shown to be a homogeneous group, which seems to indicate that there have been few introductions of the pathogen into the region.
3. The simple + duplex PCR method used for the detection of Psa has allowed the correct identification of 33 Psa strains. However, it is not specific to Pfm, since of the twenty strains that initially gave the result described for this pathovar, only two were identified as Pfm.
4. A total of 18 atypical strains were grouped in a phylogenetic tree with *P. avellanae*, *P. syringae* pv. antirrhini and a group of strains described as close to cf. Pfm.
5. The pathogenicity tests carried out on pepper gave similar results for the atypical bacteria tested, so it will be necessary to carry the same tests out on kiwifruit plants to clarify their role in the kiwifruit pathosystem.

## Figures and Tables

**Table 1:** Origin and features of *Pseudomonas syringae* isolates from the Principality of Asturias, from 2014 to 2020, that were included in this study.

Year	Site	Isolate	Origin	ID	F	L	*lsc*
2014	Langreo	LPPA 2710	leaves	Psa	−	+	C
		LPPA 2711	leaves	Psa	−	+	C
		LPPA 2712	buds	Psa	−	+	C
		LPPA 2713	leaves	Psa	−	+	C
		LPPA 2717	buds	Psa	−	+	C
		LPPA 2720	buds	Psa	−	+	C
		LPPA 2727	leaves	Psa	−	+	BC
		LPPA 2728	buds	Psa	−	+	BC
		LPPA 2729	branch	Psa	−	+	BC
		LPPA 2730	leaves	Psa	−	+	C
		LPPA 2731	leaves	Psa	−	+	BC
	Repollés/Pravia	LPPA 2714	leaves	Psa	−	+	C
		LPPA 2715	leaves	Psa	−	+	C
		LPPA 2716	leaves	Psa	−	+	C
		LPPA 2721	leaves	Psa	−	+	C
		LPPA 2754	leaves	Psa	−	+	BC
		LPPA 2755	leaves	Psa	−	+	BC
	Ibias	LPPA 2718	branch	Psa	−	+	C
	Villamayor/Piloña	LPPA 2719	buds	Ps	+	−	−
	Repollés/Pravia	LPPA 2722	leaves	Ps	+	−	C
		LPPA 2758	leaves	Ps	+	−	−
		LPPA 2759	leaves	Ps	−	−	C
	Cudillero	LPPA 2723	leaves	Ps	+	−	−
		LPPA 2724	leaves	Ps	+	−	−
		LPPA 2725	leaves	Ps	+	−	−
		LPPA 2726	leaves	Ps	+	−	−
	Santianes/Pravia	LPPA 2756	leaves	Ps	+	+	BC
		LPPA 2757	leaves	Ps	+	+	BC
		LPPA 2760	leaves	Ps	+	−	−
		LPPA 2761	leaves	Ps	+	−	C
		LPPA 2762	leaves	Ps	+	−	−
		LPPA 2763	leaves	Ps	+	−	−
		LPPA 2764	leaves	Ps	+	−	C
		LPPA 2765	leaves	Ps	+	−	C
		LPPA 2766	leaves	Ps	+	−	C
		LPPA 2767	leaves	Ps	+	−	−
		LPPA 2768	leaves	Pfm	+	+	−
		LPPA 2769	leaves	Pfm	+	+	−
2015	Repollés/Pravia	LPPA 2980	leaves	Psa	−	+	C
		LPPA 2981	leaves	Psa	−	+	BC
		LPPA 2982	leaves	Psa	−	+	C
		LPPA 2983	leaves	Psa	−	+	BC
		LPPA 2984	leaves	Psa	−	+	BC
		LPPA 2985	leaves	Psa	−	+	BC
2018	Salas	LPPA 3697	leaves	Psa	−	+	C
	Salas	LPPA 3698	leaves	Psa	−	+	C
	Salas	LPPA 3699	leaves	Psa	−	+	BC
2020	Piloña	LPPA 3700	leaves	Psa	−	+	BC
	Piloña	LPPA 3701	leaves	Psa	−	+	C
	Villaviciosa	LPPA 3702	leaves	Psa	−	+	BC
	Villaviciosa	LPPA 3703	leaves	Psa	−	+	C
	Villaviciosa	LPPA 3704	leaves	Psa	−	+	BC
	Villaviciosa	LPPA 3705	leaves	Psa	−	+	BC

LPPA: Laboratory of Phytopathology of the Principality of Asturias; ID: identified as, F: fluorescence, L: levan test, *lsc*: levansucrase genes (B, C); Psa: *P. syringae* pv. actinidiae; Pfm: *P. syringae* pv. actinidifoliorum; Ps: *P. syringae*; +: positive, −: negative.

**Table 2:** Biochemical features of the isolates under study.

Test	Psa (n = 33)%	Pfm (n = 2)%	Ps (n = 18)%
Fluorescence	0	100	94
Glucose oxidation	100	100	100
growth at 36 °C	0	50	33
Levan	100	100	11
Oxidase	0	0	0
Arginine dihydrolase	0	0	0
Tobacco	100	100	100
Oxidative	100	100	100
Esculin	30	100	100
Sucrose	100	100	100
Casein	0	0	0
Tween 80	0	0	38
Gelatin	60	100	100
Mannitol	3	0	0
Erythritol	0	0	28
Sorbitol	30	100	33
M-inositol	66	50	61
Adonitol	0	0	0
D-Tartrate	0	0	39
L-Lactate	0	0	0
Trigonelline	100	100	100
Betaine	100	100	100
Homoserine	0	0	0
Quinate	100	100	100
Xylose	100	100	100

**Table 3:** Segregating sites and nucleotide diversity of Psa + Pfm strains under study.

Gene	m	n	S	π
*gltA*	38	879	69	0.005382
*gyrB*	38	603	66	0.008049
*rpoD*	38	781	72	0.005770
*gltA* + *gyrB* + *rpoD*	38	2263	207	0.006227

m = number of sequences, n = number of positions, S = number of segregating sites, π = nucleotide diversity.

**Table 4:** Segregating sites and nucleotide diversity of atypical *P. syringae* strains under study.

Gene	m	n	S	π
*gltA*	25	703	65	0.012575
*gyrB*	25	597	81	0.025466
*rpoD*	25	792	84	0.016014
*gltA* + *gyrB* + *rpoD*	25	2092	230	0.017591

m = number of sequences, n = number of positions, S = number of segregating sites, π = nucleotide diversity.

## Data Availability

All sequences obtained in this work were deposited in GenBank; the accession numbers are in Supplementary Table S1.

## Supplementary Materials

The following supporting information can be downloaded at: https://www.mdpi.com/article/10.3390/life14020208/s1, Figure S1A–C: Phylogenetic trees of Psa and Pfm strains with *gltA*, *gyrB* and *rpoD* genes; Figure S2A–C: Phylogenetic trees of atypical strains with *gltA*, *gyrB* and *rpoD* genes; Table S1: Accession numbers of the sequences used in this work; Table S2: Estimates of evolutionary divergence between Psa and Pfm sequences; Table S3. Estimates of evolutionary divergence between atypical strain sequences.

## References

[1] (2022). MAPA. https://www.mapa.gob.es/es/estadistica/temas/estadisticas-agrarias/agricultura/superficies-producciones-anuales-cultivos/.

[2] McCann H.C., Rikkerink E.H.A., Bertels F., Fiers M., Lu A., Rees-George J., Andersen M.T., Gleave A.P., Haubold B., Wohlers M.W. (2013). Genomic analysis of the kiwifruit pathogen *Pseudomonas syringae* pv. actinidiae provides insight into the origins of an emergent plant disease. PLoS Pathog..

[3] Young J.M., Cheesmur G.J., Welham F.V., Henshall W.R. (1988). Bacterial blight of kiwifruit. Ann. Appl. Biol..

[4] Balestra G.M., Varvaro L. (1997). *Pseudomonas syringae* pv. syringae causal agent of disease on floral buds of *Actinidia deliciosa* (A. Chev) Liang et Ferguson in Italy. J. Phytopathol..

[5] González A.J., Ávila M. (2001). Disease of floral buds of kiwifruit in Spain caused by *Pseudomonas syringae*. Plant Dis..

[6] González A.J., Rodicio M.R., Mendoza M.C. (2003). Identification of an emergent and atypical *Pseudomonas viridiflava* lineage causing bacteriosis in plants of agronomic importance in a Spanish region. Appl. Environ. Microbiol..

[7] Takikawa Y., Serizawa S., Ichikawa T., Tsuyumu S., Goto M. (1989). *Pseudomonas syringae* pv. nov.: The casual bacterium of canker of kiwifruit in Japan. Ann. Phytopath. Soc. Jpn..

[8] Vanneste J.L., Yu J., Cornish D.A., Tanner D.J., Windner R., Chapman J.R., Taylor R.K., Mackay J.F., Dowlut S. (2013). Identification, virulence and distribution of two biovars of *Pseudomonas syringae* pv. actinidiae in New Zealand. Plant Dis..

[9] Cunty A., Poliakoff F., Rivoal C., Cesbron S., Fischer-Le Saux M., Lemaire C. (2015). Characterization of *Pseudomonas syringae* pv. actinidiae (Psa) isolated from France and assignment of Psa biovar 4 to a de novo pathovar: *Pseudomonas syringae* pv. actinidifoliorum pv. nov. Plant Pathol..

[10] Butler M.I., Stockwell P.A., Black M.A., Day R.C., Lamont I.L., Poulter R.T.M. (2013). *Pseudomonas syringae* pv. actinidiae from recent outbreaks of kiwifruit bacterial canker belong to different clones that originated in China. PLoS ONE.

[11] Koh Y.J., Nou I.S. (2002). DNA markers for identification of *Pseudomonas syringae* pv. actinidiae. Mol. Cells.

[12] Scortichini M. (1994). Occurrence of *Pseudomonas syringae* pv. actinidiae on kiwifruit in Italy. Plant Pathol..

[13] Ferrante P., Scortichini M. (2009). Identification of *Pseudomonas syringae* pv. actinidiae as causal agent of bacterial canker of yellow kiwifruit (*Actinidia chinensis* Planchon) in central Italy. J. Phytopathol..

[14] Bastas K.K., Karakaya A. (2012). First report of bacterial canker of kiwifruit caused by *Pseudomonas syringae* pv. actinidiae in Turkey. Plant Dis..

[15] Balestra G.M., Renzi M., Mazzaglia A. (2010). First report of bacterial canker of *Actinidia deliciosa* caused by *Pseudomonas syringae* pv. actinidiae in Portugal. New Dis. Rep..

[16] Flores O., Prince C., Nuñez M., Vallejos A., Mardones C., Yañez C., Besoain X., Bastías R. (2018). Genetic and phenotypic characterization of indole-producing isolates of *Pseudomonas syringae* pv. actinidiae obtained from Chilean kiwifruit orchards. Front. Microbiol..

[17] Vanneste J.L., Yu J., Cornish D.A., Max S., Clark G. (2011). First report of *Pseudomonas syringae* pv. actinidiae, the causal agent of bacterial canker of kiwifruit in France. Plant Dis..

[18] Abelleira A., López M.M., Peñalver J., Aguín O., Mansilla J.P., Picoaga A., García M.J. (2011). First Report of Bacterial Canker of Kiwifruit Caused by *Pseudomonas syringae* pv. actinidiae in Spain. Plant Dis..

[19] Balestra G.M., Renzi M., Mazzaglia A. (2011). First report of *Pseudomonas syringae* pv. actinidiae on kiwifruit plants in Spain. New Dis. Rep..

[20] EPPO *RS 8*. 2011, nº Article 168. https://gd.eppo.int/reporting/article/1737.

[21] Everett K.R., Taylor R.K., Romberg M.K., Rees-George J., Fullerton R.A., Vanneste J.L., Manning M.A. (2011). First report of *Pseudomonas syringae* pv. actinidiae causing kiwifruit bacterial canker in New Zealand. Australas. Plant Dis. Notes.

[22] Meparishvili G., Gorgiladze L., Sikharulidze Z., Muradashvili M., Koiava L., Dumbadze R., Jabnidze N. (2016). First report of bacterial canker of kiwifruit caused by *Pseudomonas syringae* pv. actinidiae in Georgia. Plant Dis..

[23] EPPO *RS 2*. 2014, nº Article 026. https://gd.eppo.int/reporting/article/2746.

[24] Holeva M.C., Glynos P.E., Karafla C.D. (2015). First report of bacterial canker of kiwifruit caused by *Pseudomonas syringae* pv. actinidiae in Greece. Plant Dis..

[25] Balestra G.M., Buriani G., Cellini A., Donati I., Mazzaglia A., Spinelli F. (2018). First report of *Pseudomonas syringae* pv. actinidiae on kiwifruit pollen from Argentina. Plant Dis..

[26] Marcelletti S., Ferrante P., Petriccione M., Firrao G., Scortichini M. (2011). *Pseudomonas syringae* pv. actinidiae draft genome comparisons reveal strain-specific features envolved in adaptation and virulence to *Actinidia* species. PLoS ONE.

[27] Ciarroni S., Gallipoli L., Taratufolo M.C., Butler M.I., Poulter R.T.M., Pourcel C. (2015). Development of a multiple loci variable number of tandem repeats analysis (MLVA) to unravel the intra-pathovar structure of *Pseudomonas syringae* pv. actinidiae populations worldwide. PLoS ONE.

[28] Ferrante P., Scortichini M. (2015). Redefining the global populations of *Pseudomonas syringae* pv. actinidiae based on pathogenic, molecular and phenotypic characteristics. Plant Pathol..

[29] Fujikawa T., Sawada H. (2016). Genome analysis of the kiwifruit canker pathogen *Pseudomonas syringae* pv. actinidiae biovar 5. Sci. Rep..

[30] McCann H.C., Li L., Liu Y., Li D., Pan H., Zhong C., Rikkerink E.H.A., Templeton M.D., Straub C., Colombi E. (2017). Origin and evolution of the kiwifruit canker pandemic. Genome Biol. Evol..

[31] Fujikawa T., Sawada H. (2019). Genome analysis of *Pseudomonas syringae* pv. actinidiae biovar 6, which produces the phytotoxins, phaseolotoxin and coronatine. Sci. Rep..

[32] Donati I., Buriani G., Cellini A., Mauri S., Costa G., Spinelli F. (2014). New insights on the bacterial canker of kiwifruit (*Pseudomonas syringae* pv. actinidiae). J. Berry Res..

[33] Zhao Z., Chen J., Gao X., Zhang D., Zhang J., Wen J., Qin H., Guo M., Huang L. (2019). Comparative genomics reveal pathogenicity-related loci in *Pseudomonas syringae* pv. actinidiae biovar 3. Mol. Plant Pathol..

[34] EPPO (2023). A2 List. https://www.eppo.int/ACTIVITIES/plant_quarantine/A2_list.

[35] (2013). MAPA. https://www.mapa.gob.es/es/ministerio/servicios/publicaciones/k%20agricultura_tcm30-84021.pdf.

[36] (2015). MAPA. https://www.mapa.gob.es/es/ministerio/servicios/publicaciones/f-agricultura_tcm30-83974.pdf.

[37] (2018). MAPA. https://www.mapa.gob.es/es/ministerio/servicios/publicaciones/agricultura_tcm30-521420.pdf.

[38] (2019). MAPA. https://www.mapa.gob.es/es/ministerio/servicios/publicaciones/03_i_a_agricultura_tcm30-543438.pdf.

[39] (2020). MAPA. https://www.mapa.gob.es/es/ministerio/servicios/publicaciones/capitulo-i-a-agricultura_tcm30-571824.pdf.

[40] Abelleira A., Ares A., Aguin O., Peñalver J., Morente M.C., López M.M., Sainz M.J., Mansilla J.P. (2015). Detection and characterization of *Pseudomonas syringae* pv. actinidifoliorum in kiwifruit in Spain. J. Appl Microbiol..

[41] Morán F., Marco-Noales E., Landeras E., Roselló M., Abelleira A., Gonzalez A.J., López M.M. (2021). Polyphasic analysis of isolates from kiwifruit reveal new genetic lineages of *Pseudomonas syringae* pv. actinidifoliorum look-alike. Agronomy.

[42] European and Mediterranean Plant Protection Organization (2014). PM 7/120 (1) *Pseudomonas syringae* pv. actinidiae. EPPO Bull..

[43] Gallelli A., L’Aurora A., Loreti S. (2011). Gene sequence analysis for the molecular detection of *Pseudomonas syringae* pv. actinidiae developing diagnostic protocols. J. Plant Pathol..

[44] Rees-George J., Vanneste J.L., Cornish D.A., Pushparajah I.P.S., Yu J., Templeton M.D., Everett K.R. (2010). Detection of *Pseudomonas syringae* pv. actinidiae using polymerase chain reaction (PCR) primers based on the 16S-23S rDNA intertranscribed spacer region and comparison with PCR primers based on other gene regions. Plant Pathol..

[45] Lelliott R.A., Billing E., Hayward A.C. (1966). A determinative scheme for fluorescent plant pathogenic bacteria. J. Appl. Bacteriol..

[46] Jansing H., Rudolph K. (1990). A sensitive and quick test for determination of bean seed infestation by *Pseudomonas syringae* pv. phaseolicola. Z. Pflanzenkrankh. Pflanzenschutz.

[47] Goszczynska T., Serfortein J. (1998). Milk-Tween agar, a semiselective medium for isolation and differentiation of *Pseudomonas syringae* pv. syringae, *Pseudomonas syringae* pv. phaseolicola and *Xanthomonas axonopodis* pv. phaseoli. J. Microbiol. Methods.

[48] Schaad N.W., Jones J.B., Chun W. (2001). Laboratory Guide for Identification of Plant-Pathogenic Bacteria.

[49] Bereswill S., Burgert P., Völksch B., Ullrrich M., Bender C.L., Geider K. (1994). Identification and relatedness of coronatine-producing *Pseudomonas syringae* pathovars by PCR analysis and sequence determination of the amplification products. Appl. Environ. Microbiol..

[50] Sawada H., Kanaya S., Tsuda M., Suzuki F., Azegami K., Saitou N. (2002). A phylogenomic study of the OCTase genes in *Pseudomonas syringae* pathovars: The horizontal transfer of the *argK-tox* cluster and the evolutionary history of OCTase gens on their genomes. J. Mol. Evol..

[51] Templeton M.D., Reinhardt L.A., Collyer C.A., Mitchell R.E., Cleland W.W. (2005). Kinetic analysis of the L-ornithine transcarbamoylase from *Pseudomonas savastanoi* pv. phaseolicola that is resistant to the transition state analogue (*R*)-*N^δ^*-(*N*’-Sulfodiaminophosphinyl)-L-ornithine. Biochemistry.

[52] Sorensen K.N., Kim K.H., Takemoto J.Y. (1998). PCR detection of cyclic lipodepsinonapeptide-producing *Pseudomonas syringae* pv. syringae and similarity strains. Appl. Environ. Microbiol..

[53] Bultreys A., Gheysen I. (1999). Biological and molecular detection of toxic lipodepsipeptide-producing *Pseudomonas syringae* strains and PCR identification in plants. Appl. Environ. Microbiol..

[54] Scholz-Schroeder B.K., Hutchison M.L., Grgurina I., Gross D.C. (2001). The contribution of syringopeptin and syringomycin to virulence of *Pseudomonas syringae* pv. syringae strain B301D on the basis of *sypA* and *syrB1* biosynthesis mutant analysis. Mol. Plant Microbe Interact..

[55] Li H., Ullrich M.S. (2001). Characterization and mutational analysis of three allelic *lsc* genes encoding levansucrase in *Pseudomonas syringae*. J. Bacteriol..

[56] Sarkar S.F., Guttman D.S. (2004). Evolution of the core genome of *Pseudomonas syringae*, a highly clonal, endemic plant pathogen. Appl. Environ. Microbiol..

[57] Hwang M.S.H., Morgan R.L., Sarkar S.F., Wang P.W., Guttman D.S. (2005). Phylogenetic characterization of virulence and resistance phenotypes of *Pseudomonas syringae*. Appl. Environ. Microbiol..

[58] Altschul S.F., Madden T.L., Schaffer A.A., Zhang J., Zhang Z., Miller W., Lipman D.J. (1997). Gapped BLAST and PSI-BLAST: A new generation of protein database search programs. Nucleic Acids Res..

[59] Thompson J.D., Higgins D.G., Gibson T.J. (1994). CLUSTAL W: Improving the sensitivity of progressive multiple sequence alignment through sequence weighting, position-specific gap penalties and weight matrix choice. Nucleic Acids Res..

[60] Tamura K., Stecher G., Kumar S. (2021). MEGA11: Molecular Evolutionary Genetics Analysis version 11. Mol. Biol. Evol..

[61] Bender C.L., Alarcón-Chaidez F., Gross D.C. (1999). *Pseudomonas syringae* phytotoxins: Mode of action, regulation and biosynthesis by peptide and polyketide synthetases. Microbiol. Mol. Biol. Rev..

[62] Denny T.P. (1995). Involvement of bacterial polysaccharides in plant pathogenesis. Ann. Rev. Phytopathol..

[63] Khandekar S., Srivastava A., Pletzer D., Stahl A., Ullrich M.S. (2014). The conserved upstream region of *lscB/C* determines expression of different levansucrase genes in plant pathogen *Pseudomonas syringae*. BMC Microbiol..

[64] Argudín M.A., Pérez C., Mendoza M.C., Rodicio M.R., González A.J., Méndez-Vilas A. (2006). Phenotypic and genetic diversity of fluorescent *Pseudomonas* recovered from different host plants. Modern Multidisciplinary Applied Microbiology Exploiting Microbes and Their Interactions.

[65] Yin H., Cao L., Xie M., Chen Q., Qiu G., Zhou J., Wu L., Wang D., Liu X. (2008). Bacterial diversity based on 16S rRNA and *gyrB* genes at Yinshan mine, China. Syst. Appl. Microbiol..

[66] He R., Liu P., Jia B., Xue S., Wang X., Hu J., Al Shoffe Y., Gallipoli L., Mazzaglia A., Balestra G.M. (2019). Genetic diversity of *Pseudomonas syringae* pv. actinidiae strains from different geographic regions in China. Phytopathology.

[67] Pei Y., Ma L., Zheng X., Yao K., Fu X., Chen H., Chang X., Zhang M., Gong G. (2023). Identification and genetic characterization of *Pseudomonas syringae* pv. actinidiae from kiwifruit in Sichuan, China. Plant Dis..

[68] Figueira D., Garcia E., Ares A., Tiago I., Veríssimo A., Costa J. (2020). Genetic diversity of *Pseudomonas syringae* pv. actinidiae: Seasonal and spatial population dynamics. Microorganisms.

[69] Garcia E., Moura L., Abelleira A., Aguín O., Ares A., Mansilla P. (2018). Characterization of *Pseudomonas syringae* pv. actinidiae biovar 3 on kiwifruit in north-west Portugal. J. Appl. Microbiol..

[70] Ares A., Tacão M., Figueira D., Garcia E., Costa J. (2021). Draft genome resources sequences of six *Pseudomonas syringae* pv. actinidiae strains isolated from *Actinidia chinensis* var. deliciosa leaves in Portugal. Phytopathology.

[71] Parkinson N., Bryant R., Bew J., Elphinstone J. (2011). Rapid phylogenetic identification of members of the *Pseudomonas syringae* species complex using the *rpoD* locus. Plant Pathol..

[72] Visnovsky S.B., Marroni M.V., Pushparajah S., Everett K.R., Taylor R.K., Vinatzer B.A., Pitman A.R. (2019). Using multilocus sequence analysis to distinguish pathogenic from saprotrophic strains of *Pseudomonas* from stone fruit and kiwifruit. Eur. J. Plant Pathol..

[73] Latorre B.A., Jones A.L. (1979). Evaluation of weeds and plant refuse as potential sources of inoculum of *Pseudomonas syringae* in bacterial canker of cherry. Phytopathology.

[74] Zhao Y., Damicone J.P., Bender C.L. (2002). Detection, survival, and sources of inoculum for bacterial diseases of leafy crucifers in Oklahoma. Plant Dis..

[75] Fernández-Sanz A.M., Rodicio M.R., González A.J. (2016). *Pseudomonas syringae* pv. phaseolicola isolated from weeds in bean crops fields. Lett. Appl. Microbiol..

[76] Fernández-Sanz A.M., Rodicio M.R., González A.J. (2020). Phenotypic and genotypic analysis of *Pseudomonas syringae* recovered from symptomatic beans and associated weeds in Northern Spain. Eur. J. Plant Pathol..

[77] Hirano S.S., Upper C.D. (2000). Bacteria in the leaf ecosystem with emphasis on *Pseudomonas syringae*: A pathogen, ice nucleus, and epiphyte. Microbiol. Mol. Biol. Rev..

[78] Mohr T.J., Liu H., Yan S., Morris C.E., Castillo J.A., Jelenska J., Vinatzer B.A. (2008). Naturally occurring nonpathogenic isolates of the plant pathogen *Pseudomonas syringae* lack a type III secretion system and effector gene orthologues. J. Bacteriol..

[79] Morris C.E., Sands D.C., Vanneste J.L., Montarry J., Oakley B., Guilbaud C., Glaux C. (2010). Inferring the evolutionary history of the plant pathogen *Pseudomonas syringae* from its biogeography in headwaters of rivers in north. MBIO.

[80] Frampton R., Acedo E., Young V., Chen D., Tong B., Taylor C., Easingwood R., Pitman A., Kleffmann T., Bostina M. (2015). Genome, proteome and structure of a T7-Like bacteriophage of the kiwifruit canker phytopathogen *Pseudomonas syringae* pv. actinidiae. Viruses.

[81] Pinheiro L.A.M., Pereira C., Frazão C., Balcão V.M., Almeida A. (2019). Efficiency of phage φ6 for biocontrol of *Pseudomonas syringae* pv. syringae: An in vitro preliminary study. Microorganisms.

[82] Wojtus J.K., Frampton R.A., Warring S., Hendrickson H., Fineran P.C. (2019). Genome sequence of a jumbo bacteriophage that infects the kiwifruit phytopathogen *Pseudomonas syringae* pv. actinidiae. Microbiol. Resour. Announc..

[83] Zhang H., Wu H., Xia H., Zhong C., Li L., Zeng C. (2022). Genomic characterization of two nickie-like bacteriophages that infect the kiwifruit canker phytopathogen *Pseudomonas syringae* pv. actinidiae. Arch. Virol..

[84] Warring S.L., Malone L.M., Jayaraman J., Easingwood R.A., Rigano L.A., Frampton R.A., Visnovsky S.B., Addison S.M., Hernández L., Pitman A.R. (2022). A lipopolysaccharide-dependent phage infects a pseudomonad phytopathogen and can evolve to evade phage resistance. Environ. Microbiol..

[85] Fiorillo A., Frezza D., Di Lallo G., Visconti S. (2023). A Phage Therapy model for the prevention of *Pseudomonas syringae* pv. actinidiae infection of kiwifruit plants. Plant Dis..

