# Supplementary material for: Occurrence of Pseudomonas syringae pvs. actinidiae, actinidifoliorum and Other P. syringae Strains on Kiwifruit in Northern Spain
Source: Life (Basel). 2024 Jan 31;14(2):208. doi: 10.3390/life14020208 (PMC10890144; doi:10.3390/life14020208)
Supplement: Supplementary file 1 [file life-14-00208-s001.zip › Suppl Table S1-accession numbers.pdf]

Table S1. Accession numbers of the sequences used in this work.

ISOLATE	*gyrB*	*rpoD*	*gltA*	
LPPA 2710	OR539816	OR539840	OQ919979	This study
LPPA 2711	OR539817	OR539841	OQ919980	This study
LPPA 2712	OR539818	OR539842	OQ919981	This study
LPPA 2713	OR539819	OR539843	OQ919982	This study
LPPA 2714	OR539820	OR539844	OQ919983	This study
LPPA 2715	OR539821	OR539845	OQ919984	This study
LPPA 2716	OR539822	OR539846	OQ919985	This study
LPPA 2717	OR539823	OR539847	OQ919986	This study
LPPA 2718	OR539824	OR539848	OQ919987	This study
LPPA 2719	OR644289	OR644307	OR539865	This study
LPPA 2720	OR539825	OR539849	OQ919988	This study
LPPA 2721	OR539826	OR539850	OQ919989	This study
LPPA 2722	OR644290	OR644308	OR539866	This study
LPPA 2723	OR644291	OR644309	OR539867	This study
LPPA 2724	OR644292	OR644310	OR539868	This study
LPPA 2725	OR644293	OR644311	OR539869	This study
LPPA 2726	OR644294	OR644312	OR539870	This study
LPPA 2727	OR539827	OR539851	OQ919990	This study
LPPA 2728	OR539828	OR539852	OQ919991	This study
LPPA 2729	OR539829	OR539853	OQ919992	This study
LPPA 2730	OR539830	OR539854	OQ919993	This study
LPPA 2731	OR539831	OR539855	OQ919994	This study
LPPA 2754	OR539832	OR539856	OQ919995	This study
LPPA 2755	OR539833	OR539857	OQ919996	This study
LPPA 2756	OR644295	OR644313	OR539871	This study
LPPA 2757	OR644296	OR644314	OR539872	This study
LPPA 2758	OR644297	OR644315	OR539873	This study
LPPA 2759	OR644298	OR644316	OR539874	This study
LPPA 2760	OR644299	OR644317	OR539875	This study
LPPA 2761	OR644300	OR644318	OR539876	This study
LPPA 2762	OR644301	OR644319	OR539877	This study
LPPA 2763	OR644302	OR644320	OR539878	This study
LPPA 2764	OR644303	OR644321	OR539879	This study
LPPA 2765	OR644304	OR644322	OR539864	This study
LPPA 2766	OR644305	OR644323	OR539880	This study
LPPA 2767	OR644306	OR644324	OR539881	This study
LPPA 2768	OQ884361	OQ884357	OQ884359	This study
LPPA 2769	OQ884362	OQ884358	OQ884360	This study
LPPA 2980	OR539834	OR539858	OQ919997	This study
LPPA 2981	OR539835	OR539859	OQ919998	This study
LPPA 2982	OR539836	OR539860	OQ919999	This study
LPPA 2983	OR539837	OR539861	OQ920000	This study
LPPA 2984	OR539838	OR539862	OQ920001	This study
LPPA 2985	OR539839	OR539863	OQ920002	This study
LPPA 3697	OR501175	OR504283	OR501184	This study
LPPA 3698	OR501176	OR504284	OR501185	This study
LPPA 3699	OR501177	OR504285	OR501186	This study
LPPA 3700	OR501178	OR504286	OR501187	This study
LPPA 3701	OR501179	OR504287	OR501188	This study

LPPA 3702	OR501180	OR504288	OR501189	This study
LPPA 3703	OR501181	OR504289	OR501190	This study
LPPA 3704	OR501182	OR504290	OR501191	This study
LPPA 3705	OR501183	OR504291	OR501192	This study
P. avellanae CFBP4960	KF937603	KF937700	KF937506	-
P. syringae pv. tomato CFBP 2212	JN190421	KF937693	KF937499	-
P. syringae pv. actinidifoliorum CFBP8161	KF937688	KF937785	KF937591	-
P. syringae pv. actinidiae CFBP7811	KF937605	KF937702	KF937508	-
P. syringae pv. antirrhini 126	CP126992	CP126992	CP126992	-
