# Supplementary material for: Occurrence of Pseudomonas syringae pvs. actinidiae, actinidifoliorum and Other P. syringae Strains on Kiwifruit in Northern Spain
Source: Life (Basel). 2024 Jan 31;14(2):208. doi: 10.3390/life14020208 (PMC10890144; doi:10.3390/life14020208)
Supplement: Supplementary file 1 [file life-14-00208-s001.zip › Supplementary Fig S1.pdf]

Supplementary Material

Occurrence of *Pseudomonas syringae* pvs. actinidiae, actinidifoliorum and other *P. syringae* strains on kiwifruit in Northern Spain

Figure S1. Phylogenetic trees of Psa and Pfm strains with *gltA* (S1.A), *gyrB* (S1.B) and *rpoD* (S1.C) genes

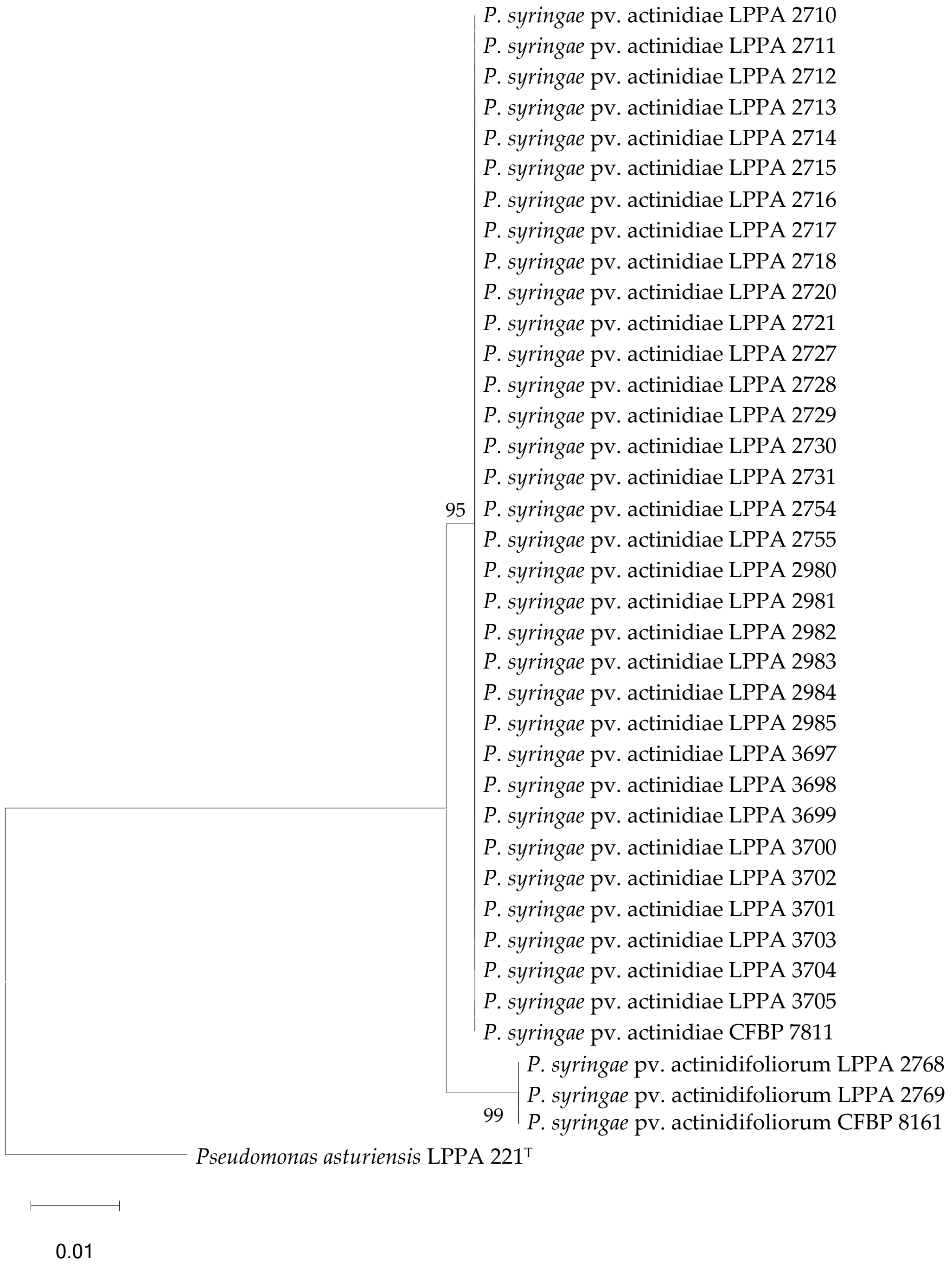

Figure S1.A. Phylogenetic tree performed with *gltA* gene, carried out using the maximum likelihood method and Tamura-Nei model. Bootstrap (>50%) is shown next to the branches. Bar, number of substitutions per site. This analysis involved 38 nucleotide sequences with 879 positions in the final dataset. Evolutionary analyses were conducted in MEGA11 [60]. *P. syringae* pv. actinidiae CFBP 7811 and *P. syringae* pv. actinidifoliorum CFBP 8161 were included as controls, and *P. asturiensis* 221^T as outgroup.

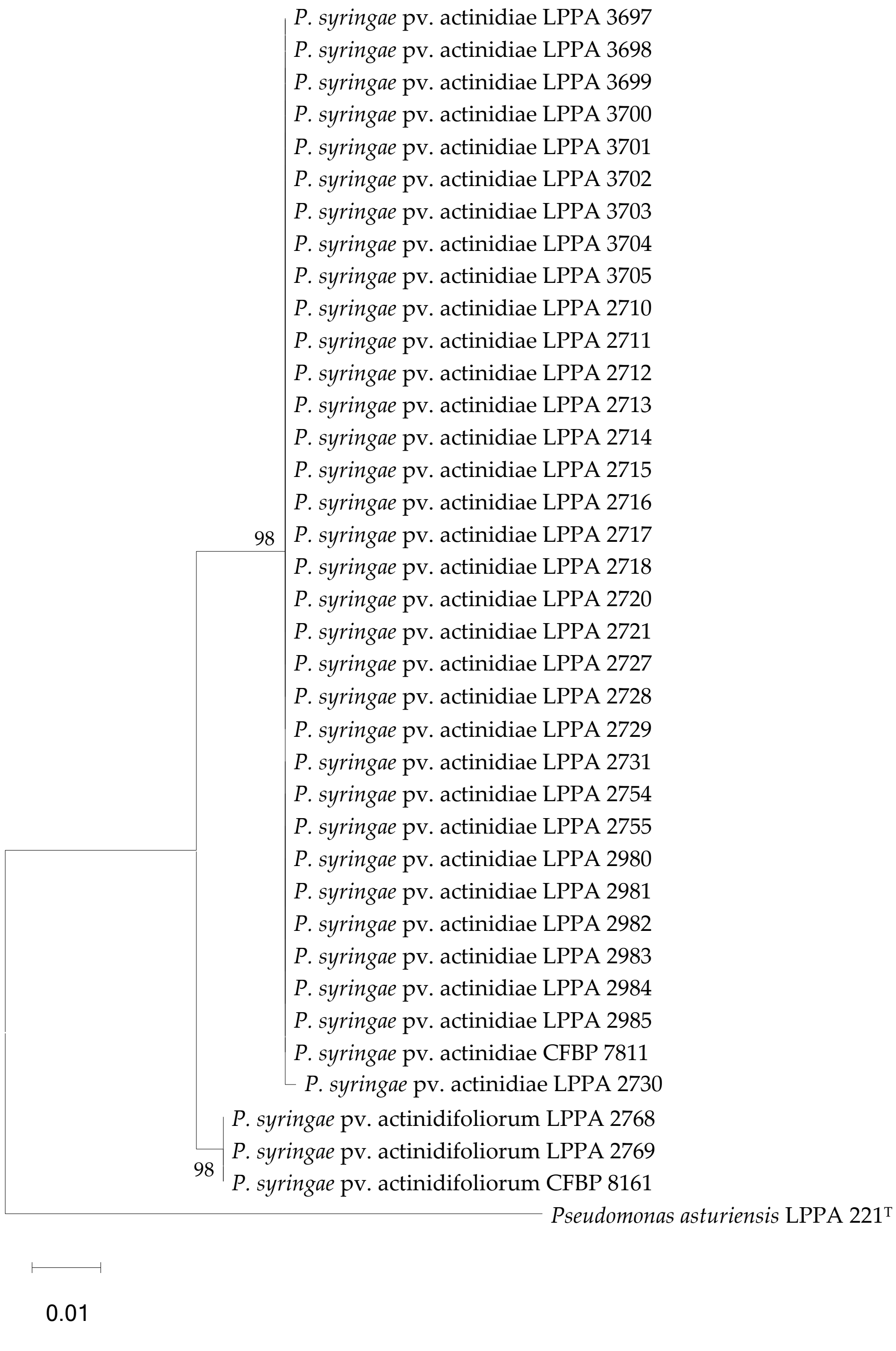

Figure S1.B. Phylogenetic tree performed with *gyrB* gene, carried out using the maximum likelihood method and Tamura-Nei model. Bootstrap (>50%) is shown next to the branches. Bar, number of substitutions per site. This analysis involved 38 nucleotide sequences with 603 positions in the final dataset. Evolutionary analyses were conducted in MEGA11 [60]. *P. syringae* pv. actinidiae CFBP 7811 and *P. syringae* pv. actinidifoliorum CFBP 8161 were included as controls, and *P. asturiensis* 221^T as outgroup.

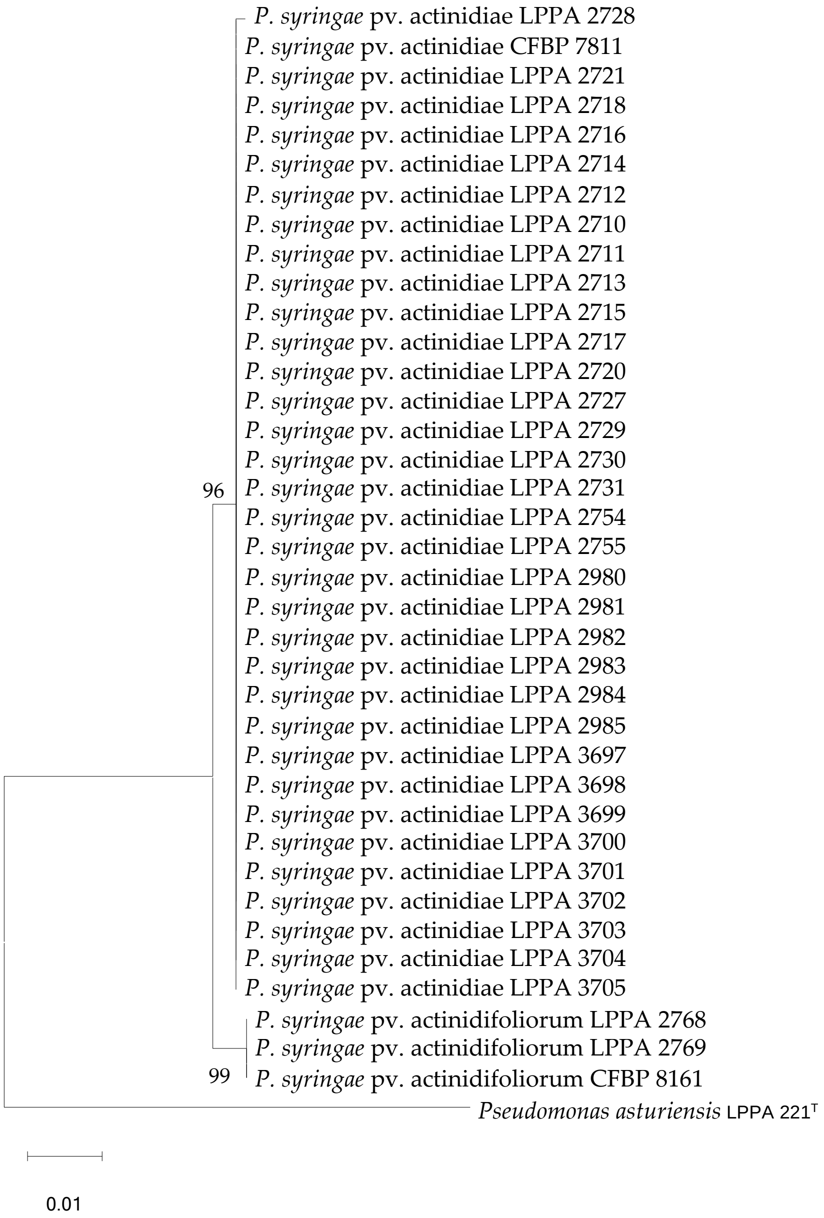

Figure S1.C. Phylogenetic tree performed with *rpoD* gene, carried out using the maximum likelihood method and Tamura-Nei model. Bootstrap (>50%) is shown next to the branches. Bar, number of substitutions per site. This analysis involved 38 nucleotide sequences with 781 positions in the final dataset. Evolutionary analyses were conducted in MEGA11 [60]. *P. syringae* pv. actinidiae CFBP 7811 and *P. syringae* pv. actinidifoliorum CFBP 8161 were included as controls, and *P. asturiensis* 221^{T} as outgroup.
