# Supplementary material for: Occurrence of Pseudomonas syringae pvs. actinidiae, actinidifoliorum and Other P. syringae Strains on Kiwifruit in Northern Spain
Source: Life (Basel). 2024 Jan 31;14(2):208. doi: 10.3390/life14020208 (PMC10890144; doi:10.3390/life14020208)
Supplement: Supplementary file 1 [file life-14-00208-s001.zip › Supplementary Fig S2.pdf]

Supplementary Material

Occurrence of *Pseudomonas syringae* pvs. actinidiae, actinidifoliorum and other *P. syringae* strains on kiwifruit in Northern Spain

Figure S2. Phylogenetic trees of atypical strains with *gltA* (S2.A), *gyrB* (S2.B) and *rpoD* (S2.C) genes

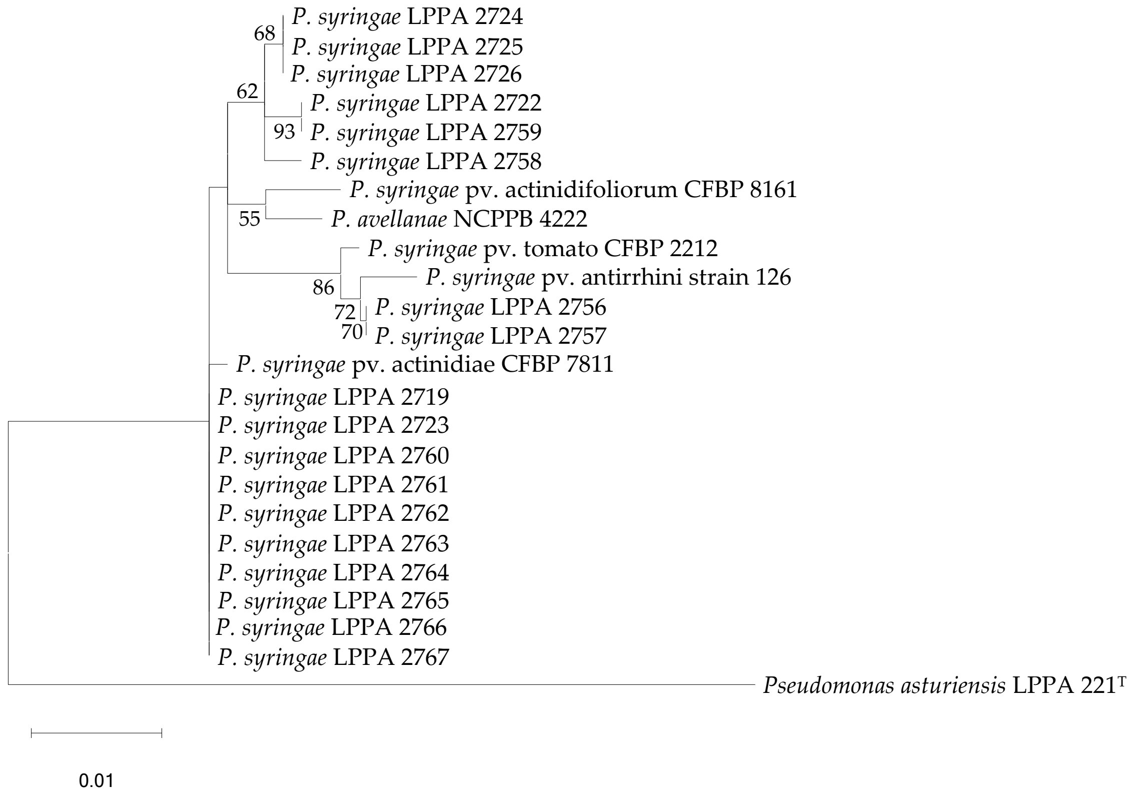

Figure S2.A. Phylogenetic tree performed with *gltA* gene, carried out using the maximum likelihood method and Tamura-Nei model. Bootstrap (>50%) is shown next to the branches. Bar, number of substitutions per site. This analysis involved 24 nucleotide sequences with 703 positions in the final dataset. Evolutionary analyses were conducted in MEGA11 [60]. *P. syringae* pv. actinidiae CFBP 7811, *P. syringae* pv. actinidifoliorum CFBP 8161, *P. syringae* pv. tomato CFBP 2212, *P. avellanae* NCPPB 4222 and *P. syringae* pv. antirrhini strain 126 were included as controls, and *P. asturiensis* 221^T as outgroup.

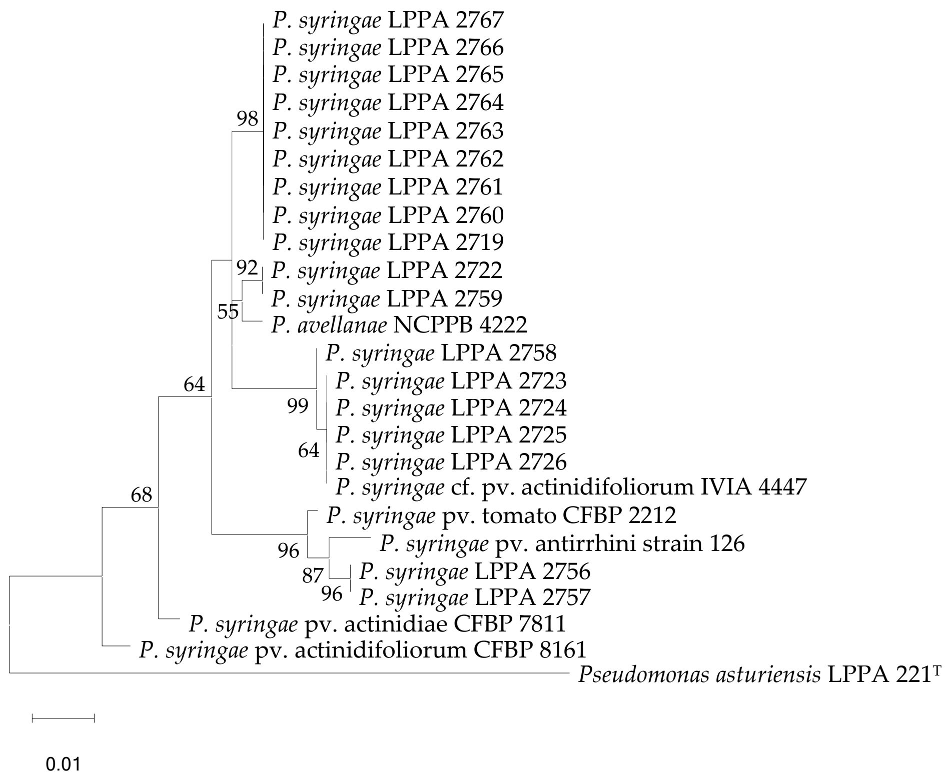

Figure S2.B. Phylogenetic tree performed with *gyrB* gene, carried out using the maximum likelihood method and Tamura-Nei model. Bootstrap (>50%) is shown next to the branches. Bar, number of substitutions per site. This analysis involved 25 nucleotide sequences with 597 positions in the final dataset. Evolutionary analyses were conducted in MEGA11 [60]. *P. syringae* pv. actinidiae CFBP 7811, *P. syringae* pv. actinidifoliorum CFBP 8161, *P. syringae* pv. tomato CFBP 2212, *P. avellanae* NCPPB 4222 and *P. syringae* pv. antirrhini strain 126 were included as controls, and *P. asturiensis* 221^{T} as outgroup.

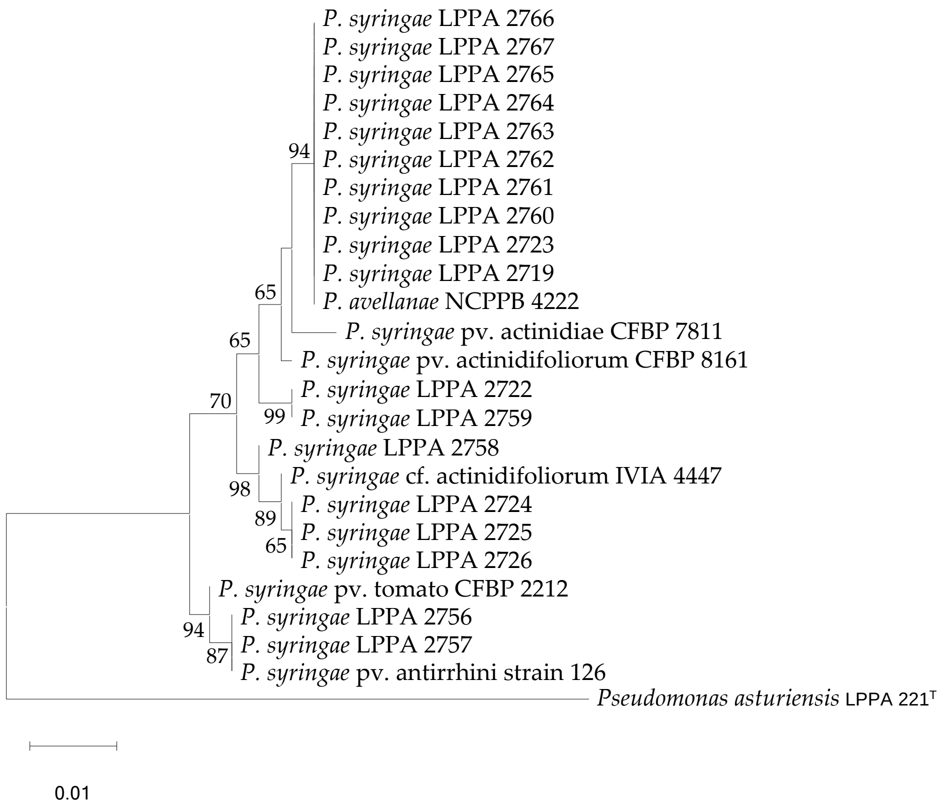

Figure S2.C. Phylogenetic tree performed with *rpoD* gene, carried out using the maximum likelihood method and Tamura-Nei model. Bootstrap (>50%) is shown next to the branches. Bar, number of substitutions per site. This analysis involved 25 nucleotide sequences with 792 positions in the final dataset. Evolutionary analyses were conducted in MEGA11 [60]. *P. syringae* pv. actinidiae CFBP 7811, *P. syringae* pv. actinidifoliorum CFBP 8161, *P. syringae* pv. tomato CFBP 2212, *P. avellanae* NCPPB 4222 and *P. syringae* pv. antirrhini strain 126 were included as controls, and *P. asturiensis* 221^T as outgroup.
